# Noninvasive diagnosis of interstitial fibrosis in chronic kidney disease: a systematic review and meta-analysis

**DOI:** 10.1080/0886022X.2024.2367021

**Published:** 2024-06-28

**Authors:** Shanshan Wan, Shiping Wang, Xinyu He, Chao Song, Jiaping Wang

**Affiliations:** a Department of Radiology, The Second Affiliated Hospital of Kunming Medical University, Kunming, China; b Department of Radiology, The Affiliated Anning First People’s Hospital of Kunming University of Science and Technology, Kunming, China

**Keywords:** Noninvasive diagnosis, chronic kidney disease (CKD), meta-analysis, renal fibrosis

## Abstract

**Rationale and objectives:**

Researchers have delved into noninvasive diagnostic methods of renal fibrosis (RF) in chronic kidney disease, including ultrasound (US), magnetic resonance imaging (MRI), and radiomics. However, the value of these diagnostic methods in the noninvasive diagnosis of RF remains contentious. Consequently, the present study aimed to systematically delineate the accuracy of the noninvasive diagnosis of RF.

**Materials and methods:**

A systematic search covering PubMed, Embase, Cochrane Library, and Web of Science databases for all data available up to 28 July 2023 was conducted for eligible studies.

**Results:**

We included 21 studies covering 4885 participants. Among them, nine studies utilized US as a noninvasive diagnostic method, eight studies used MRI, and four articles employed radiomics. The sensitivity and specificity of US for detecting RF were 0.81 (95% CI: 0.76–0.86) and 0.79 (95% CI: 0.72–0.84). The sensitivity and specificity of MRI were 0.77 (95% CI: 0.70–0.83) and 0.92 (95% CI: 0.85–0.96). The sensitivity and specificity of radiomics were 0.69 (95% CI: 0.59–0.77) and 0.78 (95% CI: 0.68–0.85).

**Conclusions:**

The current early noninvasive diagnostic methods for RF include US, MRI, and radiomics. However, this study demonstrates that US has a higher sensitivity for the detection of RF compared to MRI. Compared to US, radiomics studies based on US did not show superior advantages. Therefore, challenges still exist in the current radiomics approaches for diagnosing RF, and further exploration of optimized artificial intelligence (AI) algorithms and technologies is needed.

## 1. Introduction

Chronic kidney disease (CKD) is an irreversible and gradually progressive clinical syndrome resulting from definitive alterations in function and/or structure of the kidney. Adult patients are diagnosed with CKD when their glomerular filtration rate (GFR) stands at less than 60 mL/min/1.73 m^2^ for three months or longer, or, alternatively, evidence of renal structural injury is detected despite a GFR of over 60 mL/min/1.73 m^2^ [1]. CKD features excessive extracellular matrix deposition and chronic inflammation and is quite common worldwide. The prevalence of CKD in adults hovers around 13% in the United States and 12% in China [2]. Renal interstitial fibrosis is the pathological basis of end-stage renal disease [3].

According to von Stillfried and Triantopoulou’s research, diagnosing CKD-associated renal fibrosis (RF) is still challenging in clinical practice. Ultrasound (US) elastography, CT, and magnetic resonance imaging (MRI), have emerged as potential diagnostic methods for the noninvasive diagnosis of RF [4,5]. Imagomics, employing big data and machine learning to analyze medical image data, facilitates the development of personalized medicine and precision medicine. With the advances in technology and the deepening of application, imagomics holds the potential to furnish more dependable and precise information for disease diagnosis, treatment, and prognosis assessment. A systematic review and meta-analysis has introduced fresh perspectives for imaging diagnosis of RF. The association between interstitial fibrosis and tubular atrophy (IFTA) and the severity of CKD was closely intertwined [6]. Moderate and severe IFTA alongside glomerulosclerosis escalated the risk of declined renal function by three- and fourfold, respectively, in comparison to mild IFTA [7,8]. Existing methods for monitoring RF are currently inefficient [9]. Kidney biopsy is regarded as the gold standard for diagnosis of CKD and grading of fibrosis. The primary complications associated with native kidney biopsies predominantly encompass hemorrhagic events, which manifest as pain, hematuria, peri-nephric bleeding, resulting in a self-contained hematoma, or active bleeding requiring red blood cell transfusions or interventions to manage the hemorrhage. In severe cases, these complications may even lead to fatal outcomes [10]. Renal biopsy may cause complications, such as pain, hematomas, macroscopic hematuria, and in severe cases, bleeding or even death [11], while certain sampling bias reduces the accuracy of pathological diagnosis. Thus, noninvasive diagnostic methods for fibrosis are urgently needed to avoid these adverse events and facilitate dynamic diagnosis of patients with CKD. Therefore, exploring noninvasive diagnosis of RF has far-reaching clinical significance.

The noninvasive diagnosis of RF predominantly relies primarily on imaging methods [12,13]. In recent years, radiomics has been increasingly applied in the diagnosis and treatment of clinical diseases. Some studies have ventured into applying radiomics methods to assist in the noninvasive diagnosis of RF [14–16]. However, radiomics-based diagnosis is challenging due to the over-configuration of equipment, segmentation specificity of the region of interest (ROI), differences in extracted features, and diversity of models. Consequently, the accuracy of radiomics remains elusive. Thus, the present study aims to ascertain whether radiomics is more efficient than other noninvasive diagnostic techniques.

## 2. Methods and materials

### 2.1. Study registration

This study adhered to the PRISMA extension for Diagnostic Test Accuracy (DTA) studies. Furthermore, the study protocol has been duly registered in the PROSPERO database for systematic reviews (ID: CRD42023465028).

### 2.2. Inclusion criteria and eligibility criteria

*Inclusion criteria*:

1. Studies that used CT/MRI/US to assess the diagnostic performance in evaluating RF;
2. Studies that confirmed the staging of RF with renal biopsy as the gold standard;
3. Studies that provided data on the sensitivity and specificity of the diagnostic tests, as well as the number of patients at different stages of fibrosis, allowing the calculation of true positives (TPs), false positives (FPs), false negatives (FNs), and true negatives (TNs), thereby enabling the construction of at least one 2 × 2 test performance table for each stage of fibrosis based on threshold values.

*Exclusion criteria*:

1. Studies involving subjects with a history of kidney transplantation;
2. Unpublished conference abstracts;
3. Insufficient original data to calculate TP, FP, FN, and TN;
4. Animal experiments and basic research;
5. Studies not reported in English.

### 2.3. Data sources and search strategy

We thoroughly retrieved PubMed, Cochrane, Embase, and Web of Science databases, up to 28 July 2023. The literature search has been updated at the time of submission. MESH + free terms were adopted for searching. The search strategies are depicted in Table S1.

### 2.4. Study selection and data extraction

The retrieved records were imported into EndNote, where duplicates were identified automatically and manually. The titles or abstracts of the remaining studies were read to preliminarily select qualified original research, followed by the full-text review. The full texts of the remaining studies were downloaded and reviewed to select original research that meets the criteria for this review.

Before data extraction, a standardized data extraction spreadsheet was developed. The data to be extracted included: (1) study features: author, country, publication date, study design, and patient recruitment period; (2) patient demographics: age, number of patients, gender ratio, and body mass index (BMI); (3) indicators of performance of imaging examinations: threshold values, sensitivity, specificity, and the area under the ROC curve (AUROC); (4) technical features: SWE or MRI mode, system used, US probe, array transducer, effective number of measurements, measurement depth, and ROI; (5) histological staging of fibrosis. TP, FP, FN, and TN were computed based on the sensitivity and specificity of the diagnostic tests reported in each study.

The literature screening and data extraction were independently conducted by two researchers, Wan Shanshan (a doctor with 5 years of experience in imaging diagnosis) and Wang Jiaping (a doctor with 15 years of experience in imaging diagnosis), and the results were cross-checked upon completion. Any dissents were addressed with the assistance of a third researcher, Xinyu He.

### 2.5. Risk of bias in study

Two independent researchers leveraged the Quality Assessment of Diagnostic Accuracy Studies (QUADAS-2) tool to appraise the risk of bias in the included studies [17,18]. The QUADAS-2 consists of 14 questions in four domains (patient selection, index test, gold standard, flow, and timing) to assess two core issues: risk of bias and applicability. Each question is rated as ‘yes’, ‘no’, or ‘unclear’. A domain is considered to have a high or unclear risk of bias if at least one question within it is rated as ‘no’ or ‘unclear’. Any discrepancies between the assessments of the two researchers will be resolved by a third researcher.

### 2.6. Synthesis methods

Data analysis was executed by Stata 15.0 (StataCorp LLC, College Station, TX). A bivariate mixed-effects model was used for the meta-analysis. The meta-analysis for sensitivity and specificity was based on the diagnostic 2 × 2 tables. Nonetheless, most original studies did not provide these tables. In such cases, the diagnostic 2 × 2 tables were calculated using the following two approaches: (1) using sensitivity, specificity, and precision in combination with the number of cases; (2) extracting sensitivity and specificity based on the optimal Youden’s index and then combining them with the number of cases for calculation.

For the evaluation of the diagnostic performance of DECT for acute VFs, a bivariate mixed-effects model was employed for meta-analysis. The sensitivity, specificity, positive likelihood ratio (PLR), negative likelihood ratio (NLR), diagnostic odds ratio (DOR), and their 95% confidence interval (95% CI), and the area under the curve of comprehensive subject working characteristics (SROC) were estimated. In addition, we only pooled results from the independent validation sets during the meta-analysis of radiomics. Deek’s funnel plot was leveraged to discern publication bias, and *p* < .05 was indicative of a statistically significant difference.

## 3. Results

### 3.1. Study selection

**Figure 1.**
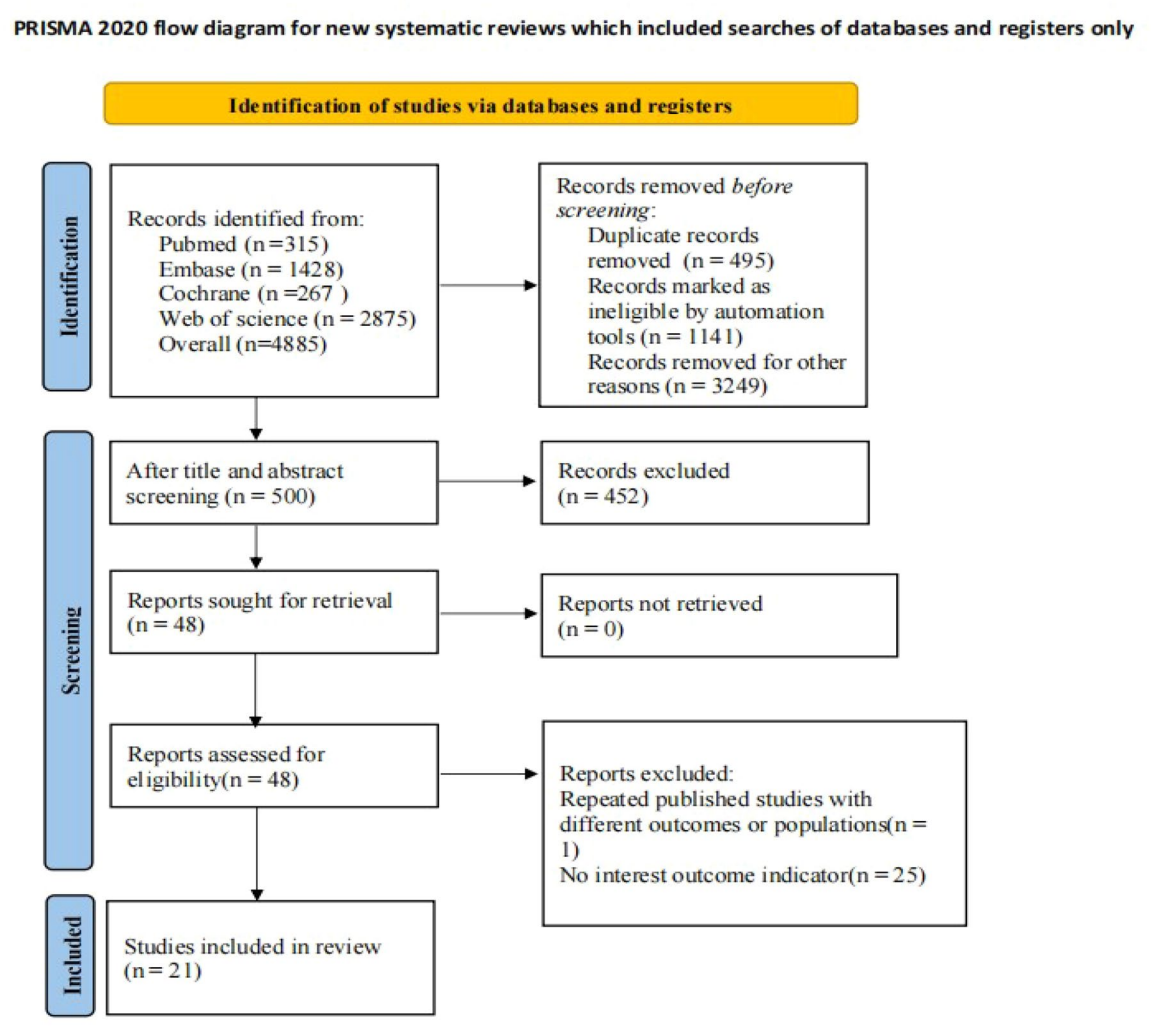
Literature screening process.

A total of 4885 articles were retrieved from the database. EndNote was used to screen the literature. After 495 duplicates were expunged, ineligible literature was eliminated through a meticulous review of abstracts and titles. Ultimately, 26 studies were deemed potentially eligible for the systematic review. Then, five studies were further excluded due to inadequate data. Finally, 21 studies [7,19–38] were incorporated into the meta-analysis. The detailed literature screening progress is depicted in Figure 1.

### 3.2. Study characteristics

A total of 21 studies with 4885 participants were incorporated into this meta-analysis. All studies adopted a single-center design. Sixteen studies [19–23,25,27–30,32–37] were based on the populations from China, and nine studies [20,22,24,25,29,32,33,37,38] used noninvasive US to assess the severity of RF. The studies indicated that US could accurately distinguish between normal and moderate-to-severe fibrosis. However, for the diagnosis of early and mild fibrosis, using SWE as an example, there is not yet a consensus among centers on the diagnostic threshold of parameters. Eight studies [7,23,26,28,31,34–36] utilized MRI techniques to detect RF, and commonly used techniques, such as diffusion-weighted imaging/apparent diffusion coefficient (DWI/ADC), intravoxel incoherent motion (IVIM), diffusion tensor imaging (DTI), and gadolinium-enhanced imaging, were compared with pathological diagnoses to assess the severity of fibrosis. The studies suggested that MRI was more effective in assessing moderate to severe fibrosis. However, the diagnostic accuracy for mild fibrosis needs to be improved. There were slight differences in MRI imaging parameters and techniques used across different centers, preventing a lateral comparison of the advantages and disadvantages of each technique. Four studies used US radiomics. It is a recently emerging artificial intelligence (AI) method for evaluating subtle differences in images, aiming at identifying imaging-based criteria for diagnosing RF that are imperceptible to the naked eye (Table 1).

**Table 1.** Fundamental features of the included literature.

No.	First author	Reference [7,19–38]	Year of publication	Type of design	Author’s country	Means of diagnosis	Number of participants	Diagnostic purpose (*f*)	Target number of cases	Total cases	Gender (male/female)	Total population age
1	Ziman Chen	20	2022	Cohort study	China	Shear wave elastography US (SWE)	All 124 patients were assigned into three groups: mildly impaired (*n* = 61), moderately impaired (*n* = 48), and severely impaired (*n* = 15).	The degree of interstitial fibrosis of the renal tubules	124	124	Male-to-female ratio of 68:56	39.67 ± 14.66
2	Liu He, MM	23	2022	Cross-sectional study	China	DTI-GBMRI	DTI-GBMRI (*n* = 92)	The degree of interstitial fibrosis of the renal tubules	92	186	53/39	52 ± 10
3	Xue Yang	22	2020	Case-control study	China	Shear wave elastography US (SWE) (YM, IF, GI)	Control group (*n* = 30) Steroid-sensitive group (*n* = 77) Steroid-resistant group (*n* = 43)	The degree of interstitial fibrosis of the renal tubules	120 (77:43)	150	77 males, 43 females	Mean age 46.9 ± 19.5 [range 23.0–68.0] years old (CG: 45.9 ± 13.0; SSG: 46.1 ± 17.3; SRG: 47.9 ± 19.2)
4	Sook Sam Leong	24	2021	Diagnostic study	Malaysia	Shear wave elastography US (SWE)	Histologic score ≤9 (*n* = 24) 10–18 (*n* = 50) ≥19 (*n* = 1)	The degree of interstitial fibrosis of the renal tubules	51	75	31 men and 44 women	Histologic score ≤9 (*n* = 24) 10–18 (*n* = 50) ≥19 (*n* = 1) Age (years) 41.33 + 16.10 46.69 + 17.93 31.00
5	Fla Viu Bob	39	2017	Prospective study	Romania	Ultrasound-based shear wave elastography (SWE)	HELTH84: DKD80	Analyze the role of point shear wave elastography (virtual touch tissue quantification using ARFI technology) in patients with diabetic kidney disease (DKD) and to determine what factors are influencing kidney shear wave speed in these patients.	49 (eGFR <60)	164	86:78	51.5 ± 17.2
6	Guanghe Cui	25	2014	Diagnostic study	China	US: virtual touch quantization (VTQ)/acoustic radiation force impulse imaging (ARFI)	Non-fibrosis (*n* = 14), mild fibrosis (*n* = 40), moderate fibrosis (*n* = 21), and severe fibrosis (*n* = 1).	The assessment of renal interstitial fibrosis	62	76	43 males and 33 females	11-75 years (40.37 ± 16.13 years)
7	Jianhua Wu	36	2021	Diagnostic study	China	Native T1 mapping with the SMART technique in assessing renal function and kidney fibrosis	CKD1 = 33, CKD2 = 26, CKD3 = 25, CKD4 = 16, CKD5 = 19	The degree of interstitial fibrosis of the renal tubules	43	138	CKD1: M19F14; CKD2: M18F8; CKD3: M8F17; CKD4	CKD1: 43 (32,55); CKD2: 46 (29,53); CKD3: 50 (44,64); CKD4: 52 (39,62); CKD5: 53 (32,66)
8	Yan Liu	37	2022	Diagnostic study	China	DKI/ADC/MRI	42	The degree of interstitial fibrosis of the renal tubules	24	42	26/16	41.3 ± 15.4
9	Chenchen Hua	28	2023	Diagnostic study	China	MRI	49 CKD, 26 healthy	The degree of interstitial fibrosis of the renal tubules	43	75	33/30	44.98 ± 14.10
10	Anthony E. Samir	29	2015	Diagnostic study	USA	SWE	45	The degree of interstitial fibrosis of the renal tubules	25	45	CKD16/9 healthy5/15	CKD61 control 34
11	Liang Wang	33	2014	Diagnostic study	China	SWE/ARFI	CDK1 = 26; CKD2 = 7	The degree of interstitial fibrosis of the renal tubules	12	45	23/22	37.1 ± 13.4
12	Iris Friedli, MS	31	2017	Diagnostic study	Switzerland	MRI	36 (8 healthy volunteers; 27 chronic kidney disease (CKD) patients)	Compare RESOLVE and ss-EPI DW sequences for the assessment of renal interstitial fibrosis.	27	36	CKD18/9; healthy not clear	53 ± 10
13	Mohammed K. Nassar	34	2021	Diagnostic study	Egypt	MRI	56 patients with CKD; 22 healthy	The degree of interstitial fibrosis of the renal tubules	56	78	23/33CKD; healthy not clear	33.8 ± 11.46
14	Chao-Gang Wei	35	2023	Diagnostic study	China	MRI	CKD = 71; HV = 40	The degree of interstitial fibrosis of the renal tubules	71	122	54/57	CKD46; HV41
15	Daniela Radulescu	32	2018	Diagnostic study	Romania	SWE/ARFI	CKD32; control 20	The degree of interstitial fibrosis of the renal tubules	32	52	31/21	CKD 62.875; control 39.05
16	Xue Yang	38	2018	Diagnostic study	China	SWV	120	The degree of interstitial fibrosis of the renal tubules	30	120	Not clear	Not clear
17	Nasr Mohamed Mohamed Osman	26	2021	Diagnostic study	DWI-MRI, ADC	DWI-MRI, ADC	60	The degree of interstitial fibrosis of the renal tubules	24	60	Not clear	Not clear
18	Ziman Chen (1)	21	2023	Diagnostic study	China	Radiomics	160	The degree of interstitial fibrosis of the renal tubules	87	160	Not clear	Not clear
19	Ziman Chen (2)	27	2023	Diagnostic study	China	Radiomics	48	The degree of interstitial fibrosis of the renal tubules	31	48	Not clear	Not clear
20	Ziman Chen (3)	19	2023	Diagnostic study	China	Radiomics	48	The degree of interstitial fibrosis of the renal tubules	24	48	Not clear	Not clear
22	Minyan Zhu	30	2021	Diagnostic study	China	Radiomics	117	The degree of interstitial fibrosis of the renal tubules	56	117	Not clear	Not clear
22	Minyan Zhu	30	2021	Diagnostic study	China	Radiomics	117	The degree of interstitial fibrosis of the renal tubules	56	117	Not clear	Not clear

### 3.3. Risk of bias studies

**Figure 2.**
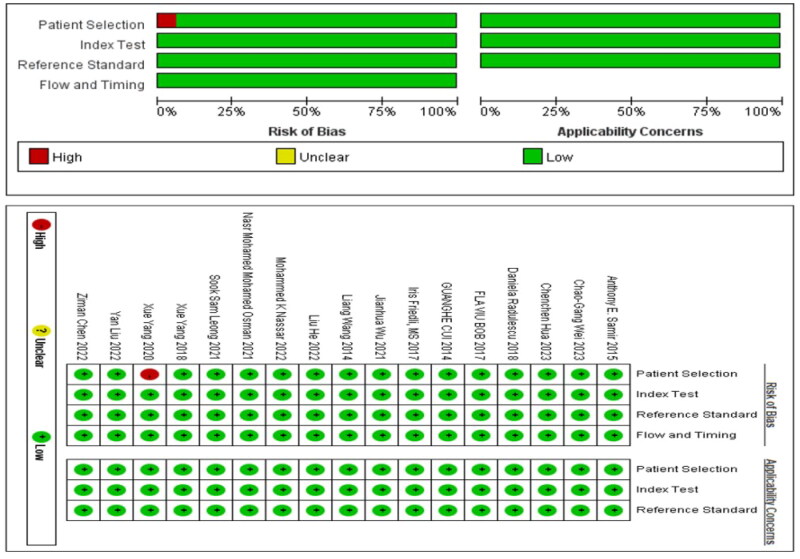
The quality assessment of 21 included studies by QUADAS-2 tool.

In the studies included in our analysis, 17 studies utilized non-radiomic approaches for diagnosing kidney fibrosis. Consequently, we employed QUADAS-2 for quality assessment. Among these, one study was a case-control study, indicating a high risk of bias in case selection (Figure 2). On the other hand, four studies employed radiomic methods for diagnosing kidney fibrosis, for which we utilized RQS for quality evaluation. Notably, none of these studies conducted repeat measurements on the same study subjects using different parameters, time points, or devices. Additionally, in terms of statistical methods, there was a lack of outcome evaluation using resampling techniques, and no external validation was conducted based on multicenter approaches. Furthermore, codes were not made publicly available. Consequently, the average score for the four studies was nine points.

### 3.4. Meta-analysis

#### 3.4.1. Ultrasound and SWE

**Figure 3.**
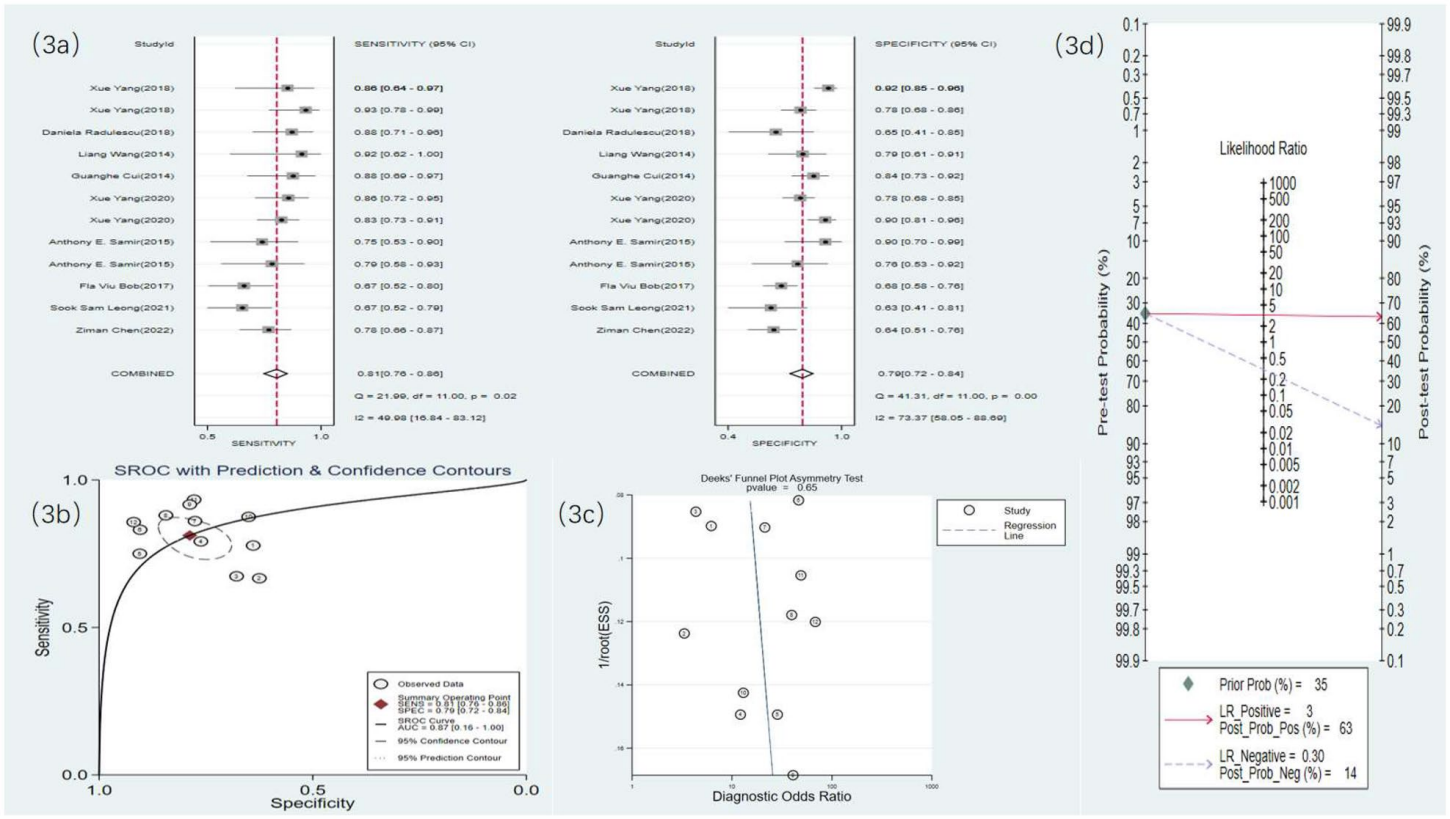
US statistical results. (a) Meta-analysis Forest plot for sensitivity and specificity of US in detecting RF. (b) SROC for meta-analysis based on US in detecting RF. (c) Deek’s funnel plot for meta-analysis based on US in detecting RF. (d) Meta-analysis line diagram based on US in detecting RF.

Nine studies used US to detect RF. The meta-analysis results showed a sensitivity of 0.81 (95% CI: 0.76–0.86), specificity of 0.79 (95% CI: 0.72–0.84), DOR of 16 (95% CI: 9–28), and an SROC curve of 0.87 (95% CI: 0.16–1.00). Deek’s funnel plot for US indicated no significant publication bias (*p* = .65). With a prior probability of 35%, the PLR was 3.8 (95% CI: 2.8–5.2) and the NLR was 0.24 (95% CI: 0.18–0.32), as shown in Figure 3.

**Figure 4.**
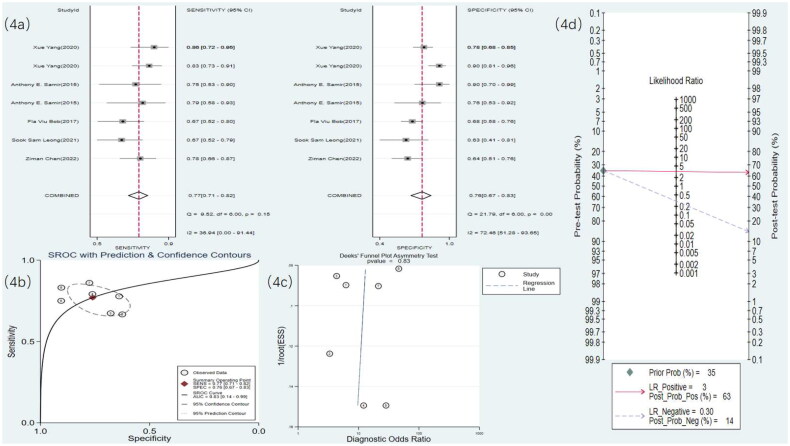
SWE statistical results. (a) Meta-analysis Forest plot for sensitivity and specificity of US in detecting RF. (b) SROC for meta-analysis based on SWE in detecting RF. (c) Deek’s funnel plot for meta-analysis based on SWE in detecting RF. (d) Meta-analysis line diagram based on SWE in detecting RF.

Seven studies used SWE to detect RF. The meta-analysis results showed a sensitivity of 0.77 (95% CI: 0.71–0.82), specificity of 0.76 (95% CI: 0.67–0.83), DOR of 11 (95% CI: 5–21), and an SROC curve of 0.83 (95% CI: 0.14–0.99). Deek’s funnel plot for SWE demonstrated no significant publication bias (*p* = .83). With a prior probability of 35%, the PLR was 3.2 (95% CI: 2.2–4.8) and the NLR was 0.30 (95% CI: 0.22–0.41), as illustrated in Figure 4.

#### 3.4.2. MRI

**Figure 5.**
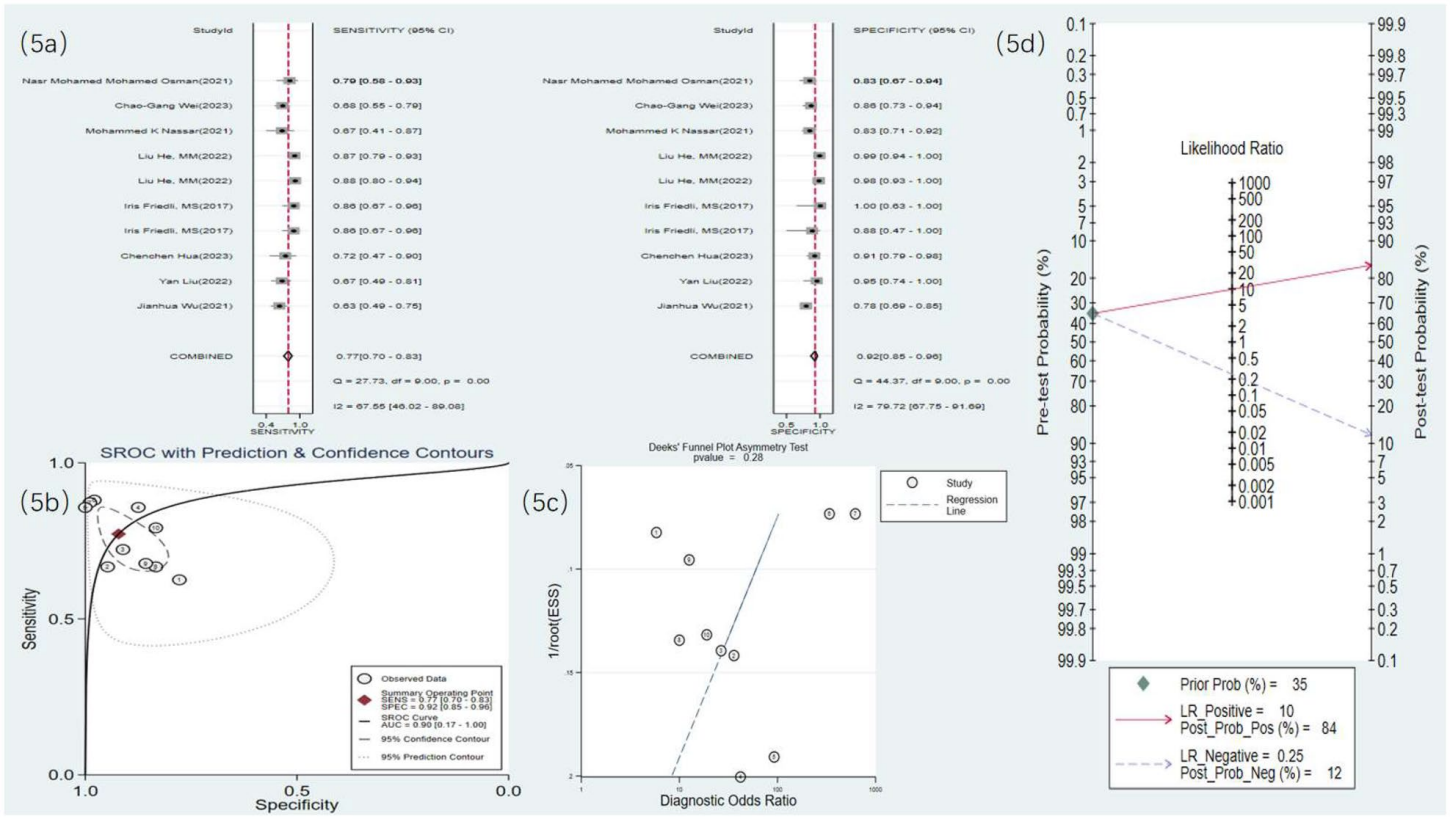
MRI statistical results. (a) Meta-analysis Forest plot for sensitivity and specificity of MRI in detecting RF. (b) SROC for meta-analysis based on MRI in detecting RF. (c) Deek’s funnel plot for meta-analysis based on MRI in detecting RF. (d) Meta-analysis line diagram based on MRI in detecting RF.

**Figure 6.**
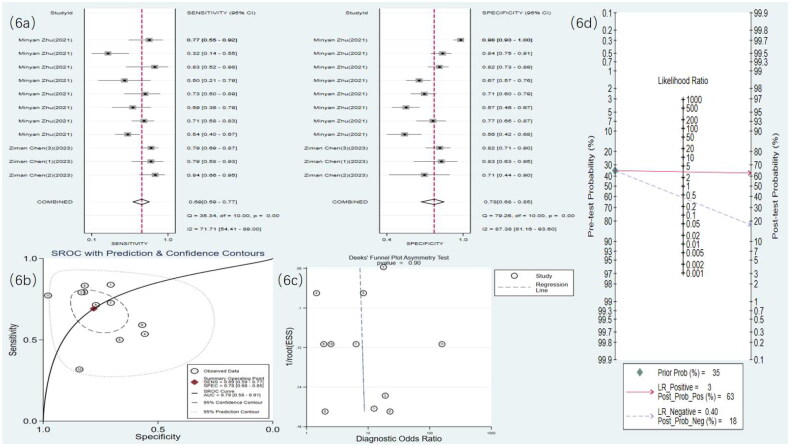
Radiomics statistical results. (a) Meta-analysis forest plot for sensitivity and specificity of radiomics in detecting RF. (b) SROC for meta-analysis based on radiomics in detecting RF. (c) Deek’s funnel plot for meta-analysis based on radiomics in detecting RF. (d) Meta-analysis line diagram based on radiomics in detecting RF

Eight studies used MRI to detect RF. The meta-analysis results showed a sensitivity of 0.77 (95% CI: 0.70–0.83), specificity of 0.92 (95% CI: 0.85–0.96), DOR of 40 (95% CI: 15–106), and an SROC curve of 0.90 (95% CI: 0.17–1.00) (Figures 5 and 6). Deek’s funnel plot for MRI revealed no significant publication bias (*p* = .28). With a prior probability of 35%, the PLR was 9.8 (95% CI: 4.9–19.7) and the NLR was 0.25 (95% CI: 0.18–0.35), as illustrated in Figure 5.

#### 3.4.3. Radiomics

Four studies [19,21,27,30] used US radiomics to detect RF. The meta-analysis results showed a sensitivity of 0.69 (95% CI: 0.59–0.77), specificity of 0.78 (95% CI: 0.68–0.85), DOR of eight (95% CI: 4–17), and an SROC curve of 0.79 (95% CI: 0.58–0.91) (Figures 5 and 6). Deek’s funnel plot for radiomics indicated no significant publication bias (*p* = .90). With a prior probability of 35%, the PLR was 3.1 (95% CI: 2.0–4.9) and the NLR was 0.40 (95% CI: 0.28–0.56), as illustrated in Figure 6.

## 4. Discussion

### 4.1. Summary of the main findings

Our meta-analysis of 21 articles revealed that nine studies utilized US, eight used MRI, and four applied radiomics to assess RF. The meta-analysis results indicated that each group was able to accurately assess the level of fibrosis, suggesting that there is no statistically significant difference in accuracy between radiomics and the direct imaging assessment methods of US and MRI. Current additional research methods include PET-CT and CT for assessing fibrosis. However, these methods are constrained by sample size limitations and the absence of comprehensive outcome indicators. Certain researchers have restricted their exploration solely to animals.

### 4.2. Diagnosis of RF with US

Routine renal US examinations and laboratory tests can reflect the patient’s condition through aspects such as kidney size and shape, cortical thickness, echo of the renal medulla, renal vascular blood flow, as well as plasma albumin, urea, and creatinine levels. These methods exhibit a certain level of subjectivity and fall short in providing a precise evaluation of the extent of renal damage. Laboratory test results for renal function are also relatively delayed; the kidneys have a strong compensatory ability, and abnormalities in renal function indicators often indicate irreversible kidney damage [39].

Previous meta-imaging studies have shown that SWE could be a potential tool for evaluating pathological changes in the kidneys. However, Cè et al. [40] reported that the relationship between USE values and eGFR in CKD patients is still elusive. Our study did not unravel a significant difference in shear wave velocity between healthy individuals and CKD patients. Perfusion alterations may play a pivotal role in the early stage of renal injury, especially in certain subgroups of patients, such as those with diabetes, due to microvascular changes. The studies by Leong et al. and Cao et al. suggested that SWE is effective in diagnosing mild and severe RF but less effective for moderate RF [24,41]. In a study to discern the performance of SWE in detecting renal parenchymal stiffness in patients with CKD [42], SWE is precise in diagnosing RF.

SWE has been shown to be an effective method for evaluating RF [19,29,30]. SWE enables real-time, noninvasive, and quantitative assessment of the renal cortical elasticity modulus in patients with primary nephrotic syndrome (PNS), and can also provide certain reference values for the efficacy evaluation and disease development and outcome of PNS patients diagnosed by biopsy after treatment [43]. SWE exhibits substantial promise in assessing RF in patients with CKD, which holds promise as a noninvasive and cost-effective imaging alternative to renal biopsy in the future. Nevertheless, the application of SWE in the kidneys remains contentious due to the influences of tissue anisotropy, viscoelasticity, and renal hemodynamics. The diversity in US systems, even those produced by the same manufacturer, may lead to variations in SWE values. It is recommended to establish more extensive datasets for each machine model to establish baseline or SWE levels indicative of different kidney stiffness. In comparison to SWE, 2D SWE with elastography allows for better selection of the ROI within the available range of SWE measurements, thereby providing more reproducible results. Given the attenuation of ARFI push pulses and tracking waves with increased skin-to-ROI depth, SWE is not suitable for renal imaging in overweight or obese patients, and variable transducer force may also influence the reproducibility and accuracy of SWE [24]. Therefore, the introduction of more advanced SWE technology, the implementation of larger-scale or multi-center studies to establish methodological standards for renal elastometry, and the establishment of reference values for normal renal elasticity are crucial to increase the reliability of SWE in the diagnosis of kidney diseases [6,32].

### 4.3. Diagnosis of RF with MRI

MRI has the advantage of noninvasive evaluation with high soft tissue resolution. The included studies used techniques such as DWI-ADC, gadolinium-enhanced imaging, IVIM, and DTI to assess the level of RF. DWI is an imaging method that studies the diffusion movement of water molecules in living tissues. IVIM addresses the limitations of traditional DWI and more accurately describes different tissue characteristics, especially in tissues with complex water molecule dynamics [44]. IVIM-DWI, based on a bi-exponential model, can separately evaluate water molecule diffusion of renal tissues, renal capillary perfusion, and tubular flow, and can more accurately reflect the microstructural changes in renal pathophysiology [45]. IVIM can reflect the density of capillaries within the tissue [46]. The decrease in the *D* value is mainly due to the increase in cell number from tissue swelling, inflammatory cell infiltration during the fibrosis process, and the deposition of collagen fibers [47,48]. The infiltration of inflammatory cells leads to an increased cellular density, causing an increase in intracellular water molecules and a decrease in extracellular water molecules, along with a decrease in extracellular water molecules due to the deposition of renal collagen fibers, causing restricted diffusion of water molecules inside and outside the cell, thereby leading to a decrease in the *D* value. During the fibrosis process in the kidney, both perfusion and diffusion decrease simultaneously, making IVIM-DWI a potential quantitative indicator for assessing RF. In previous studies, magnetic resonance elastography (MRE) technology was used to assess liver fibrosis [49,50]; recent research has found that MRE technology is also applicable to organs such as the kidneys [51]. Studies have shown that MRE can detect and assess the degree of RF. When the kidney is subjected to external forces, causing particle displacement, imaging occurs in the magnetic field. During this process, multiple alterations in the kidney occur, such as kidney stiffness, changes in renal blood flow, expansion of the collecting system, and edema. MRE can reflect changes in these indicators [52,53].

### 4.4. Diagnosis of RF with radiomics

The directionality of US speckle patterns may be detected via wavelet transformation-based radiomics characteristics, which can be utilized to differentiate between individuals with and without CKD [54]. AI-assisted medical imaging approaches provide useful information on features that are difficult for the human eye to detect for the diagnosis and treatment of CKD [55]. AI-assisted medical image analysis as a clinical support tool, and radiomics and deep learning algorithms, can enhance the early detection and prognostic evaluation of CKD. Research has evaluated the feasibility and accuracy of radiomic features of phenotype apparent diffusion coefficient (ADC) maps, which assists in the clinical classification of participants [56]. Yu et al. used CT to diagnose calcification in patients with CKD, based on a radiomic approach [57]. Radiomics is an emerging technical means of precision medicine, and the process of radiomics research is relatively complex.

The present study unveiled that the diagnostic accuracy of traditional influential diagnostics was not significantly different from that of radiomics, suggesting that the study process can be simplified and the degree of RF can be reliably assessed based on large samples of original data. Our radiomics analysis is based on a US-based model. The meta-analysis of US showed a sensitivity of 0.81 (95% CI: 0.76–0.86), and the sensitivity was 0.69 (95% CI: 0.59–0.77) for radiomics based on US. The results of US radiomics studies did not show superior sensitivity compared to direct diagnosis using US. The current US radiomics in diagnosing RF is suboptimal, underscoring the imperative to cultivate a more refined algorithm or AI assessment model for evaluating fibrosis levels in CKD.

### 4.5. Analysis of the sources of heterogeneity in different diagnostic methods

Indeed, there is heterogeneity among US, MRI, and radiomics, with moderate heterogeneity (30–60%) observed in this study. The heterogeneity in US arises from variations in diagnostic experience and procedural skills among physicians, differences in equipment diagnostic performance, and diagnostic accuracy. The diagnostic accuracy of MRI is influenced by the field strength of the equipment and the scanning techniques employed by technicians, while the accuracy of image interpretation is linked to the knowledge and diagnostic experience of physicians. Moreover, the quality of radiomics research is affected by the modeling methods utilized and the precision of the images. In this study, subgroup classification was conducted based on the characteristics of various examinations to maintain heterogeneity within a reasonable range. Factors such as the frequency of the US transducer, the strength of the MRI magnet, and the pulse sequences utilized can all impact the appearance of fibrosis. Ideally, a subgroup analysis should be performed to assess the influence of these technical factors. However, we did not obtain sufficient granular data to report this influence. Consequently, the summarized estimates of diagnostic accuracy may obscure important differences related to specific acquisition techniques, and the results of this study reflect the average values of different methods for each modality.

### 4.6. Clinical applicability analysis

US is cheaper than MRI and is more frequently utilized in clinical practice for patients CKD patients. This preference is not solely due to cost considerations but also because US-guided biopsy serves as a crucial pathological method for diagnosing CKD. Under the visualization provided by the US probe, the biopsy needle can precisely position the renal site, thereby mitigating the risks of puncture injury, bleeding, and infection by avoiding renal blood vessels and other organs. Consequently, patients diagnosed pathologically with CKD typically undergo US examination. However, MRI is not a standard diagnostic procedure for CKD in clinical practice, resulting in fewer data acquisitions. MRI, leveraging its advantages in soft tissue imaging, can integrate functional imaging and enhanced scan perfusion data to evaluate renal function, blood flow, and fibrosis levels. Nevertheless, MRI is encumbered by drawbacks, such as high costs, challenges in equipment procurement and dissemination, slow imaging speed, prolonged examination durations, the necessity for multiple sequences, and extended information analysis times, which constrain its widespread adoption and application. In recent years, however, interventional surgical treatments guided by MRI have progressively been applied in clinical practice. In the future, MRI monitoring for visualized diagnosis and treatment of CKD may become feasible, potentially necessitating updated data for analysis and evaluation. The diagnostic accuracies reported in this study represent the average values of different methods for each modality. Nonetheless, in recent years, interventional surgical treatments under MRI have gradually entered clinical practice. In the future, MRI monitoring for the visualization, diagnosis, and treatment of CKD may become possible, at which point data will be updated for analysis and evaluation. The diagnostic accuracies indicated in this study represent the average values of different methods for each modality.

### 4.7. Strengths and limitations

AI-based medical technologies are rapidly evolving, and machine learning is a sub-area of AI, broadly referring to the process of fitting predictive models to data or identifying groupings of information contained in data [58]. With the increasing use of electronic medical records, the development of patient-generated health data (PGHD) [59], and the normalization of digital pathology, there is a growing demand for the processing and analysis of large datasets and high-dimensional data. The unprecedented progress in machine learning has made the collaborative integration of AI and digital pathology a feasible reality [60]. Imaging models built using MRI and US, aside from inherent imaging factors such as imaging principles and resolution, follow a similar technical route from data annotation and feature extraction to modeling analysis. Thus, radiomics provides a means of comparing imaging data modeled from two different imaging modalities, but proving the effectiveness of certain models requires multi-center data modeling evaluations and external validations within radiomics studies. Our study is the first to comprehensively summarize the evidence for noninvasive diagnosis of RF, which offered important references for the field’s development from an evidence-based medical perspective. However, this study also faces limitations: (1) despite a comprehensive and systematic database search, the literature collected was very limited, and the imaging diagnostic devices and techniques at each center may not allow for a complete lateral comparison for evaluating RF in CKD. Conclusions drawn from a limited set of literature may restrict our interpretation of the findings. (2) In the included studies, the diagnosis of RF was confirmed by biopsy, albeit with slight variations in defining its severity. Our study discussed the diagnosis of RF through US, MRI, and radiomics; however, due to the restricted number of included studies, we were unable to delve deeper into its severity. (3) The imaging parameters for the various diagnostic methods (MRI, US, US radiomics) were not clearly noted in the articles, making it difficult to assess the consistency of the results. (4) This study included only four studies on radiomics, and conclusions drawn from this limited literature require cautious interpretation.

## 5. Conclusions

Currently, the main diagnostic methods for RF include US, MRI, and radiomics. However, our results unravel that US has a higher sensitivity for detecting RF compared to MRI. When comparing radiomics studies based on US, direct US imaging diagnosis showed better accuracy, suggesting that the current imagomics methods are not perfect and there is still a need to continue exploring more optimized AI algorithms and technologies. The comparison between radiomics studies based on US and direct US imaging diagnosis suggested that direct US imaging is more accurate, indicating that current radiomics methods are not only imperfect but also labor-intensive. Therefore, there is still a need to continue exploring more optimized AI algorithms and technologies. The accuracy differences between radiomics and direct imaging assessment methods like US and MRI are not statistically significant. Therefore, noninvasive diagnosis of RF continues to pose a substantial challenge.

In recent years, radiomics has garnered extensive interest from researchers in clinical practice. Its utility extends beyond tumor diagnosis and treatment prognosis, suggesting a promising trend for the future. In this context, very few researchers have focused on the noninvasive diagnosis of RF. The inclusion of only four radiomics studies in our research may bring some limitations in the analysis process and interpretation of results. This limitation impacts the reliability of comparisons between radiomics and other methods, such as US and MRI. The summarized estimates of diagnostic accuracy for various imaging methods may obscure important differences associated with specific acquisition techniques. Therefore, it is worth noting that our results reflect the averages of different methods for each modality. Our study has reviewed this noninvasive diagnostic approach for RF; however, further research is warranted to incorporate additional data for a more comprehensive assessment of the study’s validity and to broaden the scope of noninvasive imaging diagnostic methods for evaluating RF.

## Supplementary Material

Supplemental Material

## Data Availability

The data will not be disclosed to the public but can be shared with another research group upon a reasonable request.
