# Supplementary material for: Noninvasive diagnosis of interstitial fibrosis in chronic kidney disease: a systematic review and meta-analysis
Source: Ren Fail. 2024 Jun 28;46(2):2367021. doi: 10.1080/0886022X.2024.2367021 (PMC11216256; doi:10.1080/0886022X.2024.2367021)
Supplement: Supplemental Material [file IRNF_A_2367021_SM0326.pdf]

Table S1 Literature search strategy

1.Pubmed

Search number	Query	Results
#1	kidney fibrosis[MeSH Terms]	16048
#2	((((kidney fibrosis[Title/Abstract]) OR (Renal fibrosis[Title/Abstract])) OR (renal interstitial fibrosis[Title/Abstract])) OR (Renal vascular sclerosis[Title/Abstract])) OR (glomer-ulosclerosis[Title/Abstract])	7885
#3	machine learning[MeSH Terms]	59036
#4	(((((((((((((((((((((((machine learning[Title/Abstract]) OR (Transfer Learning[Title/Abstract])) OR (Deep learning[Title/Abstract])) OR (Ensemble Learning[Title/Abstract])) OR (artificial intelligence[Title/Abstract])) OR (random forest[Title/Abstract])) OR (neural network[Title/Abstract])) OR (neural networks[Title/Abstract])) OR (K-Nearest Neighbor[Title/Abstract])) OR (K Nearest Neighbor[Title/Abstract])) OR (Support vector machine[Title/Abstract])) OR (Gradient Boosting Machine[Title/Abstract])) OR (Nomogram[Title/Abstract])) OR (XGBoost[Title/Abstract])) OR (Adaboost[Title/Abstract])) OR (Decision tree[Title/Abstract])) OR (ResNet-50[Title/Abstract])) OR (ResNet[Title/Abstract])) OR (Naive Bayesian[Title/Abstract])) OR (Multilayer perceptron[Title/Abstract])) OR (Bayesian network[Title/Abstract])) OR (linear discriminant analysis[Title/Abstract])) OR (radiomics[Title/Abstract])) OR (Texture[Title/Abstract])) OR (radiogenomic[Title/Abstract])	312523
#5	Magnetic Resonance Imaging[MeSH Terms]	534875
#6	(((((((((((((((((Magnetic Resonance Imaging[Title/Abstract]) OR (MRI[Title/Abstract])) OR (MRIs[Title/Abstract])) OR (MR[Title/Abstract])) OR (NMR Imaging[Title/Abstract])) OR (MR Tomography[Title/Abstract])) OR (NMR Tomography[Title/Abstract])) OR (Zeugmatography[Title/Abstract])) OR (Chemical Shift Imagings[Title/Abstract])) OR (Chemical Shift Imaging[Title/Abstract])) OR (Magnetic Resonance Image[Title/Abstract])) OR (Magnetic Resonance Images[Title/Abstract])) OR (Magnetization Transfer Contrast Imaging[Title/Abstract])) OR (Proton Spin Tomography[Title/Abstract])) OR (fMRI[Title/Abstract])) OR (Functional Magnetic Resonance Imaging[Title/Abstract])) OR (Spin Echo Imaging[Title/Abstract])) OR (Spin Echo Imagings[Title/Abstract])) OR (Magnetic Resonance Imaging[MeSH Terms])	811710
#7	Ultrasonography[MeSH Terms]	492，898
#8	((((((((((((Ultrasonography[Title/Abstract]) OR (Ultrasound[Title/Abstract])) OR (Ultrasounds[Title/Abstract])) OR (Echotomography[Title/Abstract])) OR (Ultrasonic Imaging[Title/Abstract])) OR (Ultrasonographic Imaging[Title/Abstract])) OR (Ultrasonographic Imagings[Title/Abstract])) OR (Echography[Title/Abstract])) OR (elastic imaging[Title/Abstract])) OR (Ultrasonic Diagnoses[Title/Abstract])) OR (Ultrasonic Diagnosis[Title/Abstract])) OR (Computer Echotomography[Title/Abstract])) OR (Ultrasonic Tomography[Title/Abstract])) OR (Ultrasonography[MeSH Terms])	690，409
#9	Computed Tomography[MeSH Terms]	492，898

#10	#2 AND (#4 OR #6 OR #8 OR #9)	315

2.Embase

Search number	Query	Results
#1	’kidney fibrosis’/exp	17122
#2	'kidney fibrosis':ab,ti OR 'renal fibrosis':ab,ti OR 'renal interstitial fibrosis':ab,ti OR 'renal vascular sclerosis':ab,ti OR 'glomer ulosclerosis':ab,ti	11258
#3	#1 OR #2	19573
#4	'machine learning'/exp	408109
#5	'machine learning':ab,ti OR 'transfer learning':ab,ti OR 'deep learning':ab,ti OR 'ensemble learning':ab,ti OR 'artificial intelligence':ab,ti OR 'random forest':ab,ti OR 'neural network':ab,ti OR 'k-nearest neighbor':ab,ti OR 'neural networks':ab,ti OR 'k nearest neighbor':ab,ti OR 'support vector machine':ab,ti OR 'gradient boosting machine':ab,ti OR nomogram:ab,ti OR xgboost:ab,ti OR adaboost:ab,ti OR 'decision tree':ab,ti OR 'resnet 50':ab,ti OR resnet:ab,ti OR 'naive bayesian':ab,ti OR 'multilayer perceptron':ab,ti OR 'bayesian network':ab,ti OR 'linear discriminant analysis':ab,ti OR radiomics:ab,ti OR radiomic:ab,ti OR texture:ab,ti OR radiogenomic:ab,ti	356062
#6	#4 OR #5	566630
#7	'nuclear magnetic resonance imaging'/exp	1233162
#8	'magnetic resonance imaging':ab,ti OR mri:ab,ti OR mris:ab,ti OR mr:ab,ti OR 'nmr imaging':ab,ti OR 'mr tomography':ab,ti OR 'nmr tomography':ab,ti OR zeugmatography:ab,ti OR 'chemical shift imagings':ab,ti OR 'chemical shift imaging':ab,ti OR 'magnetic resonance image':ab,ti OR 'magnetic resonance images':ab,ti OR 'magnetization transfer contrast imaging':ab,ti OR 'proton spin tomography':ab,ti OR fmri:ab,ti OR 'functional magnetic resonance imaging':ab,ti OR 'spin echo imaging':ab,ti OR 'spin echo imagings':ab,ti	898068
#9	#7 OR #8	1380147
#10	'echography'/exp	1041650
#11	'ultrasonography':ab,ti OR 'ultrasound':ab,ti OR 'ultrasounds':ab,ti OR 'echotomography':ab,ti OR 'ultrasonic imaging':ab,ti OR 'ultrasonographic imaging':ab,ti OR 'ultrasonographic imagings':ab,ti OR echography:ab,ti OR 'ultrasonic diagnoses':ab,ti OR 'ultrasonic diagnosis':ab,ti OR 'computer echotomography':ab,ti OR 'ultrasonic tomography':ab,ti	602541
#12	#10 OR #11	1310156
#13	'computed tomographic colonography'/exp	4712
#14	'computed tomographic':ab,ti OR 'computed tomography':ab,ti	421256
#15	#13 OR #14	424602
#16	#6 OR #9 OR #12 OR #15	3278727
#17	#3 AND #16	1428

3.Cochrane

Search number	Query	Results
#1	(kidney fibrosis):ti,ab,kw OR (Renal fibrosis):ti,ab,kw OR (renal interstitial fibrosis):ti,ab,kw OR (Renal vascular sclerosis):ti,ab,kw	1342
#2	MeSH descriptor: [Machine Learning] explode all trees	911
#3	(machine learning):ti,ab,kw OR (Transfer Learning):ti,ab,kw OR (Deep learning):ti,ab,kw OR (Ensemble Learning):ti,ab,kw OR (artificial intelligence):ti,ab,kw	6368
#4	(random forest):ti,ab,kw OR (neural network):ti,ab,kw OR (neural networks):ti,ab,kw OR (K-Nearest Neighbor):ti,ab,kw OR (K Nearest Neighbor):ti,ab,kw	29129
#5	(K Nearest Neighbor):ti,ab,kw OR (CNN):ti,ab,kw OR (Support vector machine):ti,ab,kw OR (SVM):ti,ab,kw OR (Gradient Boosting Machine):ti,ab,kw	26216
#6	(Nomogram):ti,ab,kw OR (XGBoost):ti,ab,kw OR (Adaboost):ti,ab,kw OR (Decision tree):ti,ab,kw OR (ResNet):ti,ab,kw	2546
#7	(Naive Bayesian):ti,ab,kw OR (Multilayer perceptron):ti,ab,kw OR (Bayesian network):ti,ab,kw OR (Prediction model):ti,ab,kw OR (Risk model):ti,ab,kw	33381
#8	(linear discriminant analysis):ti,ab,kw	223
#9	#2 OR #3 OR #4 OR #5 OR #6 OR #7 OR #8	66974
#10	MeSH descriptor: [Magnetic Resonance Imaging] explode all trees	10909
#11	(Magnetic Resonance Imaging):ti,ab,kw OR (MRI):ti,ab,kw OR (MRIs):ti,ab,kw OR (MR):ti,ab,kw OR (NMR Imaging):ti,ab,kw	49505
#12	(NMR Imaging):ti,ab,kw OR (NMR Tomography):ti,ab,kw OR (Zeugmatography):ti,ab,kw OR (Chemical Shift Imagings):ti,ab,kw OR (Chemical Shift Imaging):ti,ab,kw	148
#13	(Magnetic Resonance Image):ti,ab,kw OR (Magnetic Resonance Images):ti,ab,kw OR (Magnetization Transfer Contrast Imaging):ti,ab,kw OR (Proton Spin Tomography):ti,ab,kw OR (fMRI):ti,ab,kw	10600
#14	(Functional Magnetic Resonance Imaging):ti,ab,kw OR (Spin Echo Imaging):ti,ab,kw OR (Spin Echo Imagings):ti,ab,kw	9909
#15	#10 OR #11 OR #12 OR #13 OR #14	49938
#16	MeSH descriptor: [Ultrasonography] explode all trees	17585
#17	(Ultrasonography):ti,ab,kw OR (Ultrasound):ti,ab,kw OR (Ultrasounds):ti,ab,kw OR (Echotomography):ti,ab,kw OR (Ultrasonic Imaging):ti,ab,kw	51867
#18	(Ultrasonographic Imaging):ti,ab,kw OR (elastic imaging):ti,ab,kw OR (Ultrasonographic Imagings):ti,ab,kw OR (Echography):ti,ab,kw OR (Ultrasonic Diagnoses):ti,ab,kw	8555
#19	(Ultrasonic Diagnosis):ti,ab,kw OR (Computer Echotomography):ti,ab,kw OR (Ultrasonic Tomography):ti,ab,kw	579
#20	#16 OR #17 OR #18 OR #19	59910
#21	MeSH descriptor: [Multidetector Computed Tomography] explode all trees	289
#22	(Computed Tomographic):ti,ab,kw OR (Computed Tomography):ti,ab,kw	21966
#23	#21 OR #22	21966
#24	#9 OR #15 OR #20 OR #23	182942
#25	#1 AND #24	267

4.Web of science

Search number	Query	Results
#1	kidney fibrosis (Topic) OR renal interstitial fibrosis (Topic) OR Renal fibrosis (Topic) OR glomer-ulosclerosis (Topic)	32916
#2	machine learning (Topic) OR Transfer Learning (Topic) OR Deep learning (Topic) OR Ensemble Learning (Topic) OR artificial intelligence (Topic) OR random forest (Topic) OR neural network (Topic) OR neural networks (Topic) OR K-Nearest Neighbor (Topic) OR K Nearest Neighbor (Topic) OR Support vector machine (Topic) OR Gradient Boosting Machine (Topic) OR Nomogram (Topic) OR XGBoost (Topic) OR Adaboost (Topic) OR Decision tree (Topic) OR ResNet-50 (Topic) OR ResNet (Topic) OR Naive Bayesian (Topic) OR Multilayer perceptron (Topic) OR Bayesian network (Topic) OR linear discriminant analysis (Topic) OR radiomics (Topic) OR radiomic (Topic) OR Texture (Topic) OR radiogenomic (Topic)	1653441
#3	Magnetic Resonance Imaging (Topic) OR MRI (Topic) OR MRIs (Topic) OR MR (Topic) OR NMR Imaging (Topic) OR MR Tomography (Topic) OR NMR Tomography (Topic) OR Zeugmatography (Topic) OR Chemical Shift Imagings (Topic) OR Chemical Shift Imaging (Topic) OR Magnetic Resonance Image (Topic) OR Magnetic Resonance Images (Topic) OR Magnetization Transfer Contrast Imaging (Topic) OR Proton Spin Tomography (Topic) OR fMRI (Topic) OR Functional Magnetic Resonance Imaging (Topic) OR Spin Echo Imaging (Topic) OR Spin Echo Imagings (Topic)	787285
#4	Ultrasonography (Topic) OR Ultrasound (Topic) OR Ultrasounds (Topic) OR Echotomography (Topic) OR Ultrasonic Imaging (Topic) OR Ultrasonographic Imaging (Topic) OR Ultrasonographic Imagings (Topic) OR Echography (Topic) OR Ultrasonic Diagnoses (Topic) OR Ultrasonic Diagnosis (Topic) OR Computer Echotomography (Topic) OR Ultrasonic Tomography (Topic)	529145
#5	Computed Tomographic (Topic) OR Computed Tomography (Topic)	405949
#6	#2 OR #3 OR #4 OR #5	3148086
#7	#1 AND #6	2875
